# Solid State Processing of BCZT Piezoceramics Using Ultra Low Synthesis and Sintering Temperatures

**DOI:** 10.3390/ma16030945

**Published:** 2023-01-19

**Authors:** Marzia Mureddu, José F. Bartolomé, Sonia Lopez-Esteban, Maria Dore, Stefano Enzo, Álvaro García, Sebastiano Garroni, Lorena Pardo

**Affiliations:** 1 Department of Chemical, Physical, Mathematical, and Natural Sciences, University of Sassari, Via Vienna 2, I-07100 Sassari, Italy; 2 Instituto de Ciencia de Materiales de Madrid (ICMM), Consejo Superior de Investigaciones Científicas (CSIC), c/ Sor Juna Inés de la Cruz, 3, Cantoblanco, 28049 Madrid, Spain

**Keywords:** barium calcium zirconate titanate, attrition milling, ceramics, piezoelectrics

## Abstract

Lead-free (Ba_0.92_Ca_0.08_) (Ti_0.95_ Zr_0.05_) O_3_ (BCZT) ceramics were prepared by a solid-state route (SSR) using ultra-low synthesis (700 °C/30 min and 700 °C/2 h) and sintering temperatures (from 1150 °C to 1280 °C), due to prior activation and homogenization by attrition milling of the starting high purity raw materials for 6 h before the synthesis and of the calcined powders for 3 h before the sintering. The comparison of the thermal analysis of the mixture of the starting raw materials and the same mixture after 6 h attrition milling allowed to evidence the mechanisms of activation, resulting in a significant decrease of the perovskite formation temperature (from 854 °C down to 582 °C). The secondary phases that limit the functional properties of the ceramic and their evolution with the sintering conditions were analyzed by X-ray diffraction (XRD) and scanning electron microscopy (SEM), which allowed the design of a two-step sintering method to eliminate them. A pure tetragonal BCZT perovskite phase (P4mm, c/a = 1.004) and homogeneous ceramic microstructure was obtained for synthesis at 700 °C for 2 h and sintering with the use of a two-step sintering treatment (900 °C for 3 h and 1280 °C for 6 h). The best electromechanical properties achieved were d_33_ = 455 pC/N, k_p_ = 35%, Q_m_ = 155.

## 1. Introduction

Ferroelectric materials are a model of multifunctionality and new applications of complex compositions with perovskite-type and related crystal structures [1] are being investigated currently in the emerging energy fields of harvesting [2] and photovoltaic conversion [3] or those based on the magnetodielectric effect [4]. In the consolidated field of piezoelectric sensors, actuators, motors and other devices using electromechanical transduction, replacing the market-dominating PZT (Lead Zirconium Titanate)-based tridimensional perovskite has been one of the most investigated materials research topics over the last two decades [5,6]. In this respect, one of the most promising complex oxides with an alternative, lead-free composition, which respects the human health and protects the environment, is represented by the pseudo-ternary system BaTiO_3_-BaZrO_3_-CaTiO_3_ [7], commonly known as BCZT (Barium Calcium Zirconate Titanate). BCZT has lower density than PZT (below 6 g/cm^3^) and piezoelectric coefficient, d_33_,like the best hard PZT ceramics (up to 620 pC/N [8]). An advantage of BCZT in comparison with PZT is the lower volatility of raw materials, while the main drawback is represented by the higher synthesis (up to 1350 °C) and sintering temperatures (up to 1500 °C) required for its processing by the solid-state route [9,10,11,12,13]. A recent review shows that, in these materials, dielectric and piezoelectric properties are closely related to an optimized ceramic microstructure (i.e., grain size, microporosity, etc.), that depends essentially on the fabrication process [14]. Furthermore, both raw powder particle size and sintered ceramic grain size play a crucial role in the optimization of the BCZT piezoceramics properties [15]. 

Although some efforts have been paid to the reduction of the processing temperatures by using chemical methods of synthesis [16,17,18], the use of a high thermal budget currently remains as a compulsory condition to obtain high piezoelectric coefficients in BCZT by the solid-state route [19]. BCZT is one of the most refractory lead-free piezoelectric materials, as it is also well-known for the unmodified barium titanate. This means that, when the solid-state route is used, the synthesis of the compound is usually carried at or above 1300 °C, for example in the seminal work [8]. More severe synthesis conditions are not unusual in the literature, for example, 1350 °C for 15 h in oxygen atmosphere [20]. There are a few works in which the synthesis is conducted at low conditions of 1250 °C for 2 h [21]. Only when more reactive powders are used, such as a previously mechanosynthesized powder by energetic milling in planetary mill for 12 h or sol-gel derived powders, very-low synthesis conditions of 900 °C for 1 h [22] or 1000 °C for 6 h [17], respectively, are reported.

As for the sintering conditions used by solid-state route, we can currently find reports of severe thermal treatments as, for example, 1450–1500 °C in the mentioned seminal work [8] or 1500 °C for 4 h in air, when using and additional mixing for 8 h after synthesis [23] or 1450 °C for 8 h in air [24]. Even when using synthesized sol-gel powder, the sintering was reported at 1420 °C for 6 h when the powder was ball-milled for 16 h after synthesis [17] or 1500 °C for 10 h [25].

However, ball-milling has proven to be effective in reducing the processing temperatures of electroceramics [26,27]. What is more, attrition ball-milling is very effective in homogenizing the particle size of raw materials, which leads to the decrease in the synthesis temperature, as previously reported for other lead-free materials [28,29]. Furthermore, another attrition ball-milling after synthesis seems to be necessary to decrease the sintering temperature, by reducing the initial particle size of calcined powders [30]. This is because the driving force for sintering is inversely proportional to the particle size, as previously reported [31]. However, in the case of BCZT piezoceramics, attrition milling for solid-state processing studies are lacking.

Within such context, the present investigation aims to analyze the effect of attrition ball-milling on the processing temperatures, the microstructural and piezoelectric properties of the here fabricated high sensitivity BCZT ceramics using the solid-state route.

## 2. Materials and Methods

### 2.1. Materials

Ceramic powders of nominal composition (Ba_0.92_ Ca_0.08_) (Ti_0.95_ Zr_0.05_) O_3_ [32] (abbreviated as BCZT) were prepared by a solid-state route starting from a stoichiometric mixture of BaCO_3_ (Merck, Darmstadt, Germany, >99%), CaCO_3_ (Sigma Aldrich, St. Louis, MO, USA, >99%), TiO_2_ (Merck, of nominal purity >99% composed of 85 wt.% of anatase and 15 wt.% of rutile) and ZrO_2_ (Tosoh, Tokyo, Japan, monoclinic polymorph >99%). 

### 2.2. Powder and Ceramics Processing

The raw materials were dried at 80 °C overnight, weighted according to the nominal composition indicated above and, finally, mixed. To activate these raw materials, they were mechanically treated by attrition ball milling (BM). For this process, to prevent contamination, a stainless-steel vial internally coated with Teflon^®^ was chosen. Then, the mixed raw materials were milled for 6 h at 700 rpm in isopropanol as liquid medium with 3Y-TZP balls (d = 3 mm) with a Ball to Powder weight Ratio (BPR) of 30, according to previous adopted approaches [28,29,33].

The as-obtained slurry was then transferred into a beaker and heated in an oven at 80 °C to eliminate the solvent.

Thermal characterization was conducted by a TG-DSC Q600 TA Instrument. Samples of mixed raw materials and ball-milled powders for 6 h were annealed from room temperature to 1100 °C with a heating ramp of 5 °C/min. 

The ball-milled powders were calcined in an electrical furnace, in air, inside a crucible covered with a lid. The calcined powders were ground to obtain a fine particulate material.

The influence of a second attrition ball milling on the ceramic microstructure was evaluated. For this purpose, (i) one fraction of the batch previously calcined and ground was directly sieved down to 50 µm (1 BM: one step milling process) and (ii) other fraction of the batch was attrition ball-milled again for 3 h within the same vial formerly used and was subsequently dried and sieved (2 BM: two-steps milling process).

Disc-shape pellets from each type of powder were obtained by uniaxial pressing (3 min 1.2 tons). Likewise, the discs of both 1 BM and 2 BM powders were sintered under the same conditions. Different sintering times and temperatures were explored using a heating rate of 3 °C/min. The bulk densities were determined by the geometric method. 

### 2.3. Structural and Microstructural Characterization

Structural investigations were conducted using a Bruker D8TT Advance diffractometer with a Cu anode (λ = 1.54178 Å) working at 40 kV and 30 mA with a graphite monochromator in a step-scanning mode from 20° to 60° (2Ɵ°), using 0.07° as step size and 2 s of integration time. For the sake of determination of secondary phases, more detailed patterns were acquired with 0.07° step and 4 s. Ceramic microstructure characterization of the fresh fracture surface of the pellets have been accomplished using a scanning electron microscopy (SEM G2 pro; Phenom, Thermo Fisher Scientific, Waltham, MA, USA, operated with a beam voltage of 5 kV).

### 2.4. Dielectric and Electromechanical Characterization

In order to measure electric properties, pellets of approximately 12 mm diameter were reduced in thickness by polishing to a typically 1mm thickness. Silver paste was attached on both surfaces of the thin disks and sintered at 400 °C for 1 h. After that, the conductivity of the electrodes, the DC resistance of the disk and the dielectric permittivity and losses at 1 kHz of the samples were measured at room temperature. Samples were poled in thickness under 10–15 kV.cm^−1^ at 40 °C for 1 h in a silicone oil bath, followed by field cooling (FC) to room temperature for the piezoelectric characterization.

The quasi-static d_33_ piezoelectric charge coefficient, which characterizes the sensor performance of the ceramic in the poling field direction, was measured with a Berlincourt d_33_-meter at 100 Hz. Complex impedance as a function of the frequency was measured with an impedance analyzer (HP 4192A-LF) at the radial extensional resonance of the thickness poled thin disks. The impedance was plotted as Resistance(R) and Conductance (G) vs. frequency plots. The related piezoelectric, dielectric and elastic material coefficients, including all the losses, were determined using the software for automatic iterative analysis of R and G vs. frequency curves [34]. The residuals for these reconstructed R and G peaks to the experimental ones, quantified by the regression factor (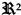), accounts for the validity of the material model for the resonance mode. The closer is the model to the experimental curves; the closer is 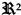 to 1. For the planar mode, the complex material coefficients (P* = P′ − iP′′) directly determined in this analysis are the piezoelectric charge coefficient, d_31_, the dielectric permittivity, ε^T^_33_, and the elastic compliances, s^E^_11_ and s^E^_12_. Additionally, a few other material coefficients are determined by the software from those, using well known relationships. These allow us to analyze the performances of the ceramic as generator (piezoelectric voltage coefficient g_31_) and as energy transducer (electromechanical coupling factors (K_p_,K_31_)and frequency number (N_p_ = f_s_(kHz). D(mm), where D is the diameter of the disk)). Losses can be expressed for each material complex coefficient as loss tangent factor (tanδ = P′′/P′), commonly used for the dielectric coefficients, or as a quality factor (Q = P′/P′′), commonly used for the elastic coefficients.

## 3. Results and Discussion

### 3.1. Processing and Characterization of the Powders 

#### 3.1.1. Thermal Analysis of the Powders

**Figure 1:**
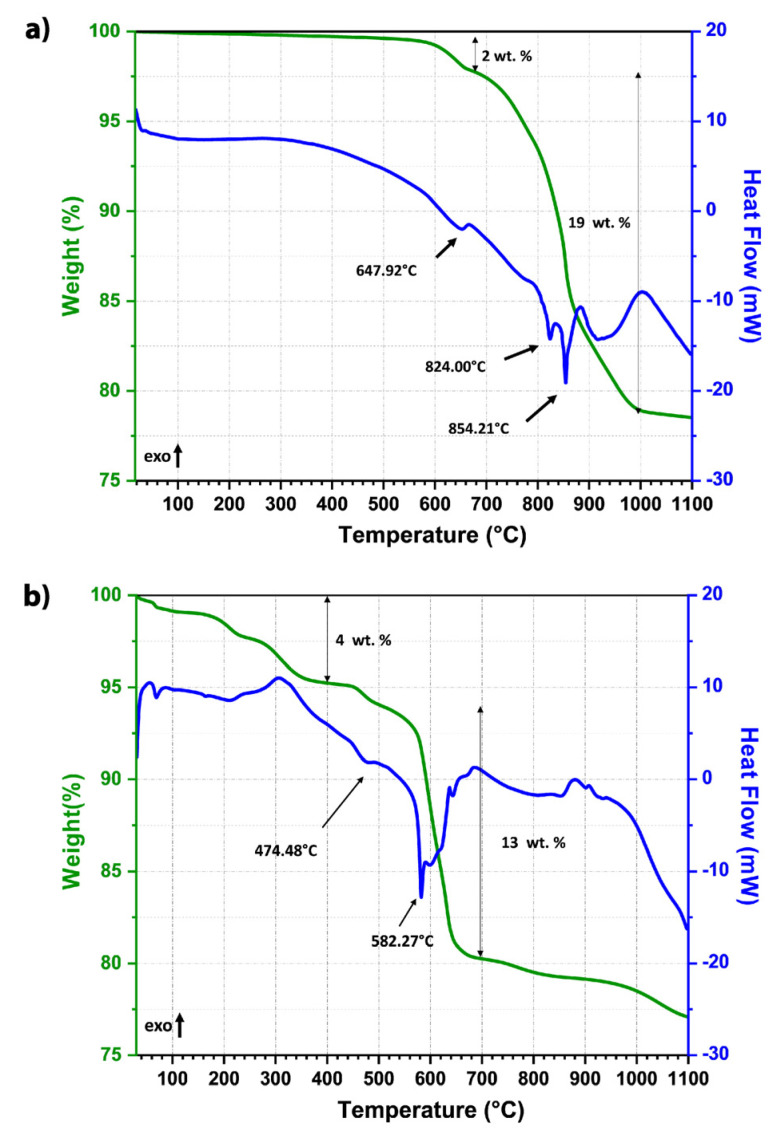
Thermal analysis (green solid line is weight of powder and blue solid line is heat flow) of mixed raw materials before (**a**) and after (**b**) attrition milling treatment for 6 h.

The influence of the mechanical processing on the activation of the starting raw materials, has been evaluated by Thermogravimetry and Differential Scanning Calorimetry (TG-DSC) measurements reported in Figure 1. The weight loss process of BCZT mixed raw materials, as shown in Figure 1a, can be summarized in two main stages. At the first stage a weight loss of 2% is observed (green solid line) between 600 and 700 °C and it is associated with an endothermic peak in the corresponding DSC profile (blue solid line). This thermal event could be correlated with the decomposition of CaCO_3_ to CaO and CO_2_. 

The second weight loss of around 19% occurred between 700 and 1000 °C and coincides with two endothermic peaks. According to the literature, the first sharp endothermic peak at ∼824 °C, could be due to the reversible phase transformation α-BaCO_3_→β-BaCO_3_, while the second one, peaked 854 °C, is related to the decomposition of carbonates and formation of perovskite BCZT [35,36]. The broad exothermic peak around 1000 °C can then be ascribable to the crystallization of BCZT powders. The powder milled for 6 h, presents interesting differences in terms of TG-DSC profiles, as showed in Figure 1b. A first weight loss of 4% in the temperature range of 300–400 °C can be related with the evaporation of the organic compounds formed during the milling treatment performed using isopropanol. The endothermic peak associated with the first decomposition of calcium carbonates appeared at lower temperature of 474 °C, if compared with those of the unmilled system. Interestingly, the main weight loss of 13% related to the endothermic events, which lead to the formation of BCZT phase, occurred between 500 °C and 700 °C, temperatures significantly lower than those achieved for the mixed raw materials. 

To confirm the results of the DSC/TGA analysis, different synthesis treatments have been performed on the ball-milled powders at the different characteristic temperatures of the milled powder. 

#### 3.1.2. X-ray Diffraction of the Powders

**Figure 2:**
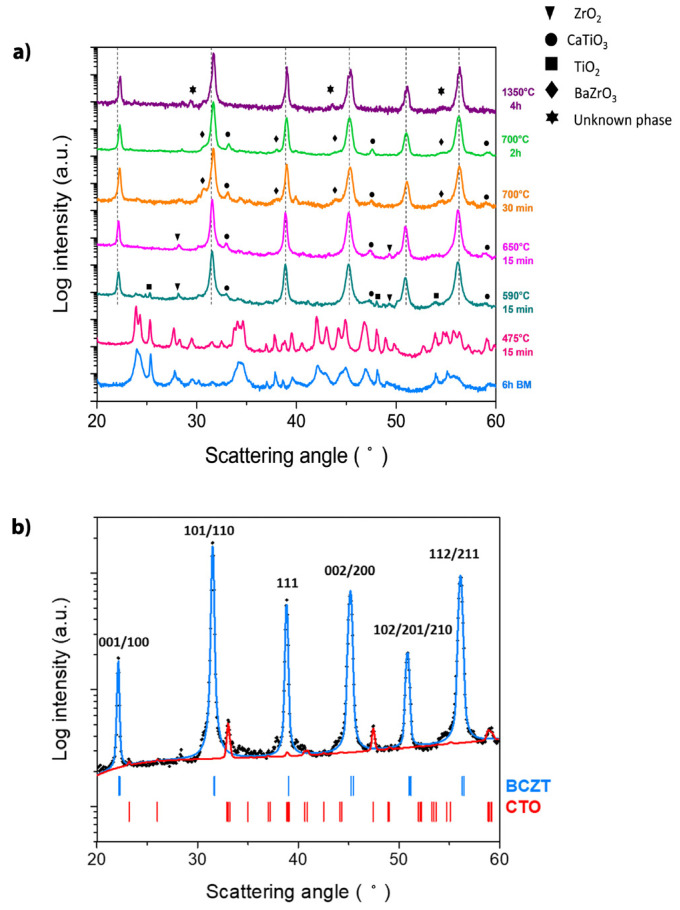
(**a**) XRD diffraction patterns of the powder ball-milled for 6 h and calcined under variable time and temperature. Symbols indicate the secondary phases that accompany the main Barium Calcium Zirconium Titanate (BCZT) perovskite. Vertical dotted lines indicate the main BCZT perovskite reflections that are marked with the Miller indexes for the tetragonal prototype. (**b**) Rietveld analysis of the calcined powder at 700 °C for 2 h, in which both BCZT and Calcium Titanate (CTO) stick patterns are shown.

The evolution of the crystalline phases with the synthesis temperature has been investigated by using XRD analysis (Figure 2) showing logarithmic scale intensities to highlight the occurrence of secondary weak phases that can escape the common analysis in the linear intensity domain, as the fraction of the BCZT perovskite is always estimated as being higher than 95% vol. The bottom pattern, corresponding to mixture of the raw materials after 6 h milling, shows a mixture of orthorhombic Witherite BaCO_3_ (Pncm, a = 5.2926 Å, b = 8.947 Å 1, c = 6.4309 Å, Vc = 304.52 Å^3^, not far from the value of 303.80 Å^3^ reported in the literature), with an amount of ca 70.0 wt.% The residual 30.0 wt.% of the pattern was mainly TiO_2_ in the anatase tetragonal form. There are also traces coincident with some peaks of monoclinic ZrO_2_ and tetragonal rutile of TiO_2_, but their appreciation is deteriorated by the noise of data. In such phase, the analysis CaCO_3_ is missing, which was included originally under the form of Calcite. To explain this, notice that the orthorhombic aragonite polymorph of CaCO_3_ is reported in the literature with the same space group of witherite and having lattice parameters a = 4.9652 Å; b = 7.9636 Å; and c = 5.7484 Å, respectively.

The second pattern from the bottom of Figure 2, corresponding to the milled powder treated at 475 °C, refers again to a mixture of Witherite and anatase in similar proportion 70.0 wt.% and 30.0 wt.%, where the lattice parameters for the orthorhombic phase evaluated this time are a = 5.3094 Å; b = 8.9053 Å and c = 6.4444 Å, Vc = 304.70 Å^3^, respectively. The differences observed in the lattice parameters reported here may be ascribed to the incorporation of ions into the orthorhombic dominant lattice of pure Witherite, which is partially destabilized with creation of various kinds of defects. Furthermore, the sharper peaks observed with respect to the as-milled powder are due to a growth process of the diffracting domain size, accompanied by a reduced degree of lattice disorder, affected by the thermal treatment conducted. 

The main perovskite of BCZT is formed after treatment at 590 °C, as shown by the third pattern from the bottom of Figure 2a (green curve). This temperature corresponds well with the main endothermic peak of DSC analysis and the main weight loss of TGA, at 582 °C, in Figure 1b. At this temperature some residual raw materials (TiO_2_ and ZrO_2_), and a secondary phase (CaTiO_3_) can also be observed. 

By increasing the temperature to 650 °C, the small amount of TiO_2_ cannot be observed anymore, but weak peaks attributable to monoclinic ZrO_2_ and CaTiO_3_ are still observed. The XRD analysis of the powder treated at 700 °C for 30 min (orange curve) shows weak shoulders marked with ◊ suggesting the formation of a secondary cubic compound, which might correspond to BaZrO_3_ derived from ZrO_2_ as previously reported [22,37]. However, the 2θ location of such shoulders occurs at values significantly different from those reported in the literature (JCPDS 6-0399), leading to a cubic lattice parameter a = 4.12 Å (to compare with the known value 4.18 Å). Moreover, another intermediate compound virtually disappears when increasing the dwell time to 2 h (light green curve). 

Increasing the synthesis temperature to very high temperature (1350 °C) leads to the mentioned huge weight loss and the undesirable hard sintered powder agglomerates. After treatment at 1350 °C, the powder seems not to be either a single-phase BCZT, although it does not contain CaTiO_3_ and has a higher distortion than that obtained at 590 °C (c = 4.0234 Å, a = 4.0020 Å, c/a = 1.005).

The reduction of the raw materials particle size leads to a decrease in synthesis temperature due to the creation of new surfaces and higher number of contact points between the particles. Moreover, the optimization and the particle refinement of the carbonates during their decomposition play a key role in the reduction of synthesis temperature to the formation of the BCZT ceramic by solid-state route [38].

**Figure 3:**
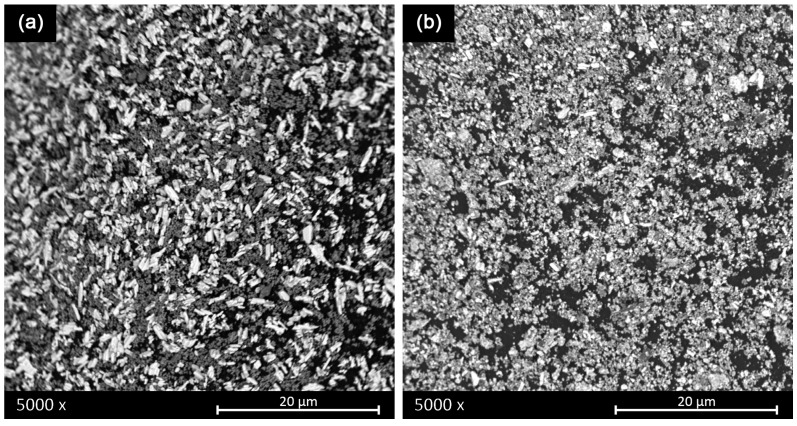
Shows the SEM micrographs of (**a**) the mixed raw materials and (**b**) the same mixture after activation by particles size reduction and homogeneization using attrition ball-milling and attrition ball-milled powders.

As mentioned above, high-energy attrition ball milling is very effective in reducing the particle size of the raw materials. This is evident by comparing SEM images of unmilled powders (0 h) and attrition ball-milled powders for 6 h, as shown in Figure 3.

### 3.2. Processing and Characterization of the Sintered Disks

#### 3.2.1. X-ray Diffraction of the Sintered Disks

**Figure 4:**
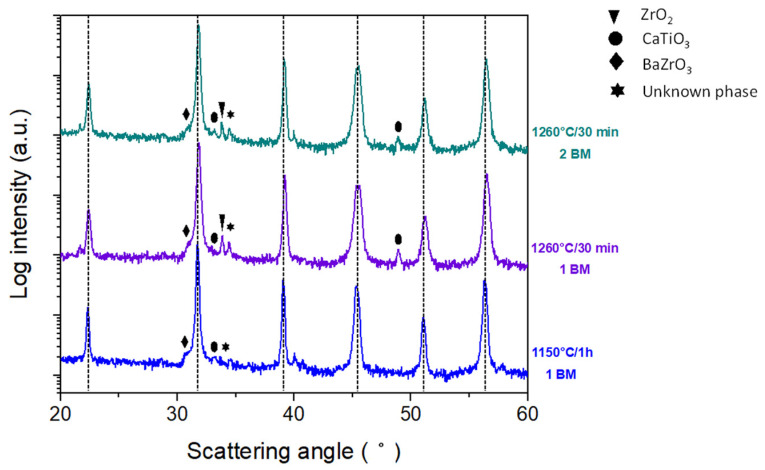
XRD diffraction patterns of the sintered ceramics, after synthesis at 700 °C for 30 min, at different sintering times and temperatures using a single sintering plateau, with and without a second ball milling treatment after synthesis (2 BM and 1 BM, respectively). Vertical dotted lines indicate the main BCZT perovskite reflections.

The comparison of the patterns for both ceramics sintered at 1260 °C for 30 min with (2 BM) and without (1 BM) a ball milling after synthesis reveals little differences from the structural point of view (Figure 4). However, in these two groups of samples, the density increased slightly from the sample 1 BM (4.5 g/cc) to the sample 2 BM (4.6 g/cc).

**Figure 5:**
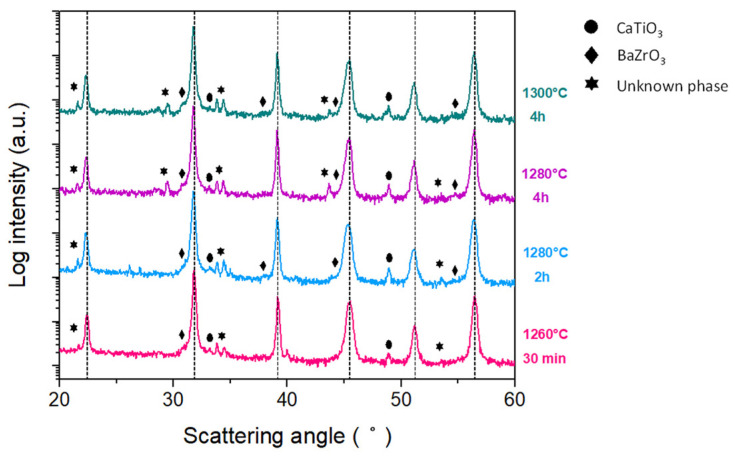
XRD diffraction patterns of the ceramics obtained by calcinating the powders at 700 °C for 30 min and subsequent sintering of the pellets at different sintering times and temperatures, using a single sintering plateau. Vertical dotted lines indicate the main BCZT perovskite reflections. The secondary phases that accompany the main BCZT perovskite are marked.

When using a one-step sintering method (Figure 5), the increase in the temperature or time of the sintering treatment fails to reduce the content of second phases (barium zirconate, titanium oxide, zirconium oxide and calcium titanate together with other, most probably transient, phases, such as BaCaTiO_4_). This can be due to the incomplete incorporation of Zr^4+^ into the B crystallographic site in this secondary phase [19].

Previously [39], an enhanced sintering behavior was observed in barium titanate by treatment of the aqueous processed powder at 950 °C for 15 h or performing such a treatment during heating of the samples up to the sintering temperature. Based on this, in light of the thermal analysis of the calcined and milled powders where the main exothermic peak can be observed at 900 °C (Figure 1b), a set of two-step sintering experiments was conducted at 900 °C and different sintering times ranging between 1 and 4 h. The final sintering temperature was 1280 °C. The starting powder was synthesized at 700 °C for 2 h, aiming to enhance its homogeneity.

**Figure 6:**
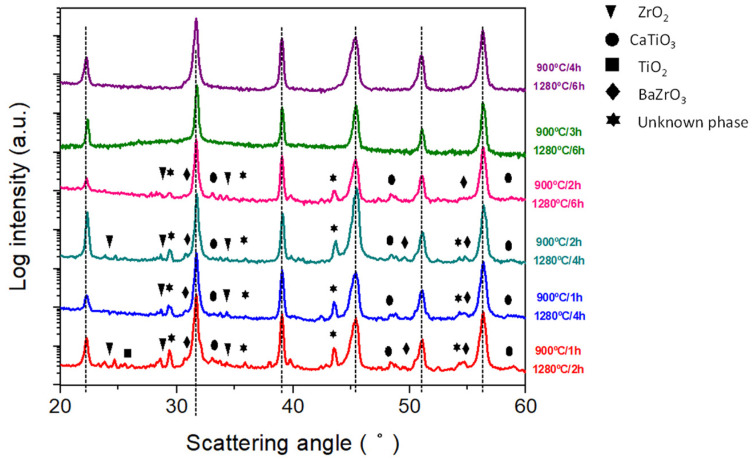
XRD diffraction patterns of the sintered ceramics after synthesis at 700 °C 2 h using a two-step sintering method at different sintering times and temperatures. Vertical dotted lines indicate the main BCZT perovskite reflections. Symbols indicate the secondary phases that accompany the main BCZT perovskite.

As shown in Figure 6, after an appropriate two-step sintering treatment (900 °C/3 h and 1280 °C/6 h) the secondary phases, such as raw materials and intermediate compounds, volatilize or incorporate into the main BCZT perovskite.

As mentioned at the beginning, the interesting piezoelectric properties are ascribable to the main tetragonal phase ascertained and characterized. Accordingly, Table 1 shows the lattice parameters and the ratio c/a of the main perovskite phase.

#### 3.2.2. SEM Analysis of the Sintered Ceramics

**Figure 7:**
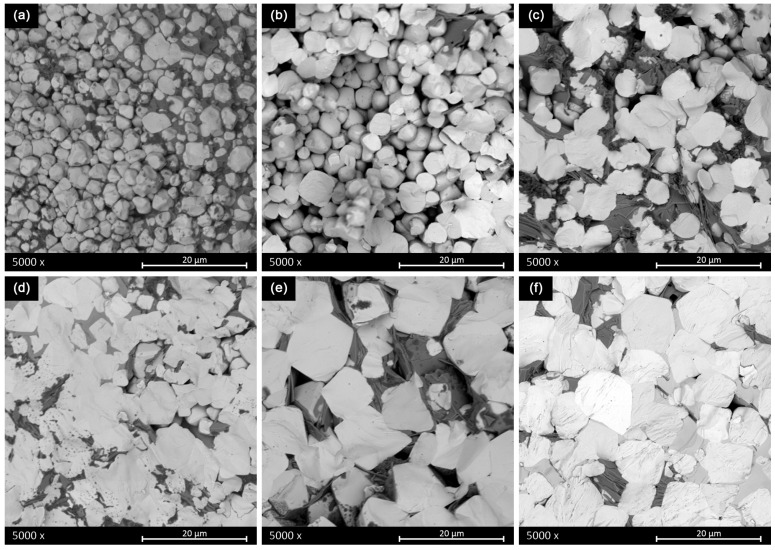
Scanning electron micrographs of the sintered ceramics obtained from powder calcined at 700 °C for 30 min, and subsequent sintering of the pellets at different sintering times and temperatures, using a single sintering plateau: (**a**) 1150 °C for 1 h, 1 BM (**b**) 1260 °C for 30 min, 1 BM, (**c**) 1260 °C for 30 min, 2 BM (**d**) 1280 °C for 2 h, 2 BM, (**e**) 1280 °C for 4, 2 BM and (**f**) 1300 °C for 4 h, 2 BM.

Figure 7 shows the SEM micrographs of the fractured surfaces of the sintered ceramics obtained from powder calcined at 700 °C for 30 min and subsequent sintering of the pellets at different sintering times and temperatures using a one-step sintering method.

By comparing Figure 7a,b, it is evident that insufficient sintering temperature does not allow adequate grain growth. Furthermore, the samples (a) and (b) show an intergranular fracture, due to weak grain boundaries. 

The comparison of images in Figure 7b,c of sintered ceramics under the same conditions proved that the second attrition ball milling promotes the grain growth using the same temperature. As the temperature increases, the grain size also increases and as the time (at the same temperature) increases, the homogeneity of grain size increases.

The underlaying mechanism of these development is the enhancement of the calcined powder reactivity through the dispersion of agglomerates of submicron size particles, which promotes higher contact points where the mass transport needed for the sintering is activated, confirmed by SEM analysis. By comparing images in Figure 7c,f, it is clear that the increase of the sintering time and temperature fails to eliminate the secondary phases, even though grain growth is promoted (from 2 μm to about 12 μm) and the samples reveal a majoritarian transgranular fracture, due to stronger grain boundaries. Figure 7f shows a degraded sintering stage with intragrain porosity. Therefore, single-step sintering is not effective in eliminating the secondary phases. 

**Figure 8:**
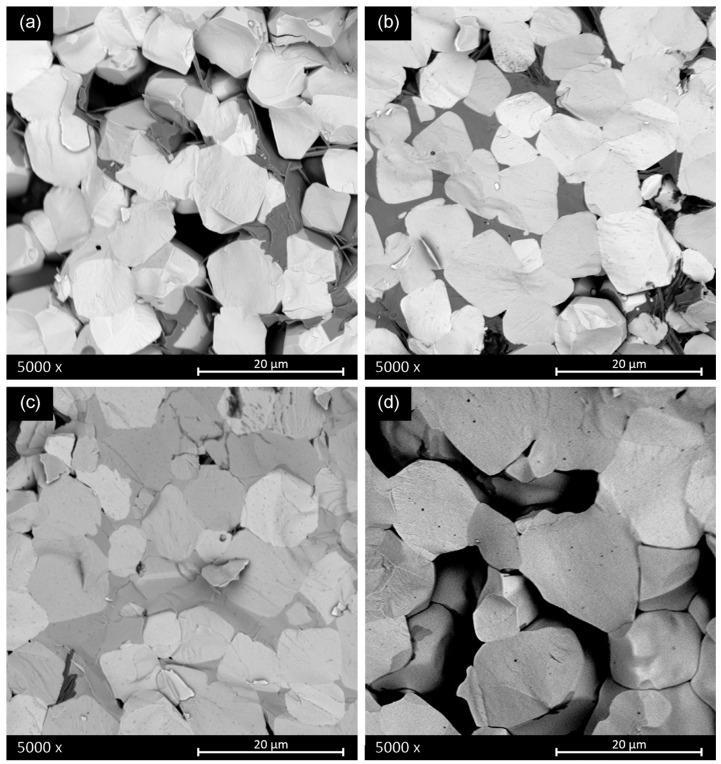
Scanning electron micrographs of the sintered ceramics obtained from powder calcined at 700 °C and subsequent sintering of the pellets at different sintering times and temperatures using a two-step sintering method: (**a**) 900 °C for 1 h/1280 °C for 2 h (**b**) 900 °C for 1 h/1280 °C for 4 h (**c**) 900 °C for 2 h/1280 °C for 6 h and (**d**) 900 °C for 4 h/1280 °C for 6 h.

Figure 8 shows the SEM micrographs of the fractured surfaces of sintered ceramics obtained from powder calcined at 700 °C for 2 h and subsequent sintering of the pellets at different sintering times and temperatures using a two-step sintering method.

As shown in Figure 8, an appropriate two-step sintering treatment promotes grain growth and allows the amount of crystallized secondary lamellar phase to decrease, resulting in a nearly pure BCZT perovskite phase. The preliminary sintering step stabilizes the powder phase and promotes the decrease of secondary phases. During this step, the porosity created by the elimination of the secondary phase promotes adequate grain growth during the second sintering step (from about 6 μm to 20 μm).

### 3.3. Electrical Characterization

The electrical characterization was accomplished together with the structural one and with the determination of grain and porosity of the sintered ceramics. This allowed us to evaluate the structural and ceramic microstructural features that influenced the poling process of the sintered ceramics and determined the final material properties. All this provided integrated feedback for the decision taking stage for the next steps of the processing of the materials, aiming to get the optimal electromechanical activity of these high sensitivity piezoceramics.

Table 2 shows the preliminary electrical characterization of the ceramics sintered from powder calcined with the thermal budget of 700 °C for 30 min. The optimum sintering conditions for getting the highest density, resistivity and d_33_ piezo coefficient together with the lowest dielectric losses corresponds to a 2 BM ceramic sintered at 1280 °C for 4 h. These sintering conditions are well below those reported in the literature for solid-state route of processing, as explained in the introduction of this manuscript, to produce dense solid-state BCZT ceramics. This temperature reduction depends on the reactivity of the synthesized powder at these ultra-low conditions and reactivated by the second ball milling that aims to increase the grain size (Figure 7) and density of the sintered ceramic (Table 2). One could think that the simple action of increasing the sintering temperature could lead to a sample with better overall performance. Contrarily, the XRD pattern of Figure 5 and the SEM micrograph Figure 7 of the sample sintered with this higher thermal budget show that the ceramic microstructure suffers a degradation. It shows lower density and intergranular porosity and, besides, that the secondary phases are not eliminated, which results in lowering the overall performance. 

The relatively low performance of the mentioned best sample in this set of experiments, specifically the low d_33_ piezo coefficient (189 pC/N) reveals the key importance of the intergranular secondary phases (Figure 5 and Figure 7) in the ceramic functionality. Table 3 shows the preliminary electrical characterization of the ceramics sintered from powder calcined at 700 °C for 2 h and milling after synthesis using a two-step sintering method and the optimum final sintering temperature determined previously. The first thing that calls the attention to these results is that whereas the increase of the time at 1280 °C (from 2 to 6 h) results in an increase of density, the increase of the time at 900 °C (from 2 to 4 h) results in a decrease of the secondary phase. A simple discussion of the properties in terms of the variation of the density is not applicable here. The reason could be that there are two competitive driving forces in the thermal evolution of the microstructure. On the one hand, secondary phases can be observed in samples with only one sintering step (Figure 5 and Figure 7) and the first stages of sintering with two-step (Figure 6 and Figure 8) as pale liquid phase and crystallized lamella. These phases either volatilize or incorporate into the main BCZT grains from the intragrain volume, mainly at 900 °C, leaving a residual porosity. On the other hand, the reduction of the porosity and increase of grain size, mainly at 1280 °C, is more effective as the time at this temperature increases. However, the elimination of the secondary phases seems to be completed only after 3 h at 900 °C (Figure 7) and it seems to create an increasing porosity as the time at 900 °C increases. The densification procedure, at the last step of 1280 °C for 6 h, is limited when the sample is at 900 °C for 3 h and 4 h and their density decreases, though their porosity is constituted by micron-size porosity, which is homogeneously distributed. Additionally, these ceramics have a single phase BCZT composition (Figure 6 and Table 1) with homogeneous grain size above 10 μm (Figure 8). Despite the ultra-low conditions of synthesis and sintering used for their processing, their optimized structure and microstructure resulted in the achievement of high sensitivity BCZT ceramics (d_33_ piezo coefficient > 400 pC/N). 

Resonance measurements at the planar mode of the electrically induced electromechanical resonance were made to complete the characterization of selected ceramic samples with important piezoelectric performance (d_33_ > 150 pC/N) with the calculation of piezo-elastic-dielectric coefficients including all losses by the iterative method (Table 4). In addition to this, and for the sake of comparison, a low density and fine grain size ceramic (<5 μm) prepared without a second ball milling from powder sintered at 700 °C for 30 min (Figure 5) and a dense ceramic (4.90 g/cc) prepared from powder synthesized at 1350 °C for 4 h and sintered at 1450 °C were also analyzed.

The fine grain ceramic with low density and secondary phases sintered at 1150 °C for 1 h is characterized by a low k_p_ electromechanical coupling coefficient, low d_31_ and g_31_ coefficients, together with a low permittivity at resonance $\varepsilon \prime_{33}^{T}$ and a high mechanical quality factor Q_m_. Contrarily, the ceramic sintered at 1450 °C (d_33_ = 210 pC/N) has high coupling and piezoelectric coefficients, higher $\varepsilon \prime_{33}^{T}$ and lower Q_m_.

Among all ceramics prepared with ultra-low synthesis and sintering treatment and a second ball milling after synthesis, the performance of those obtained with one sintering step compares well from the mechanical and dielectric permittivity points of view, but they have lower coupling factor and piezoelectric coefficients.

Table 5 shows a comparison of the piezoelectric coefficient (d_33_) of different compositions of BCZT ceramics prepared by different synthesis methods, such as solid-state and other synthesis routes and with Li-doping, a well-known agent to enhance sinterability. The composition more commonly reported in the BCZT ternary system is the (Ba_0.85_Ca_0.15_)(Zr_0.10_Ti_0.90_)O_3_ (BCZT-1510) while the nominal composition under study in the present work is the (Ba_0.92_Ca_0.08_)(Zr_0.05_Ti_0.95_)O_3_ (BCZT-0805) of which scarce information exists in the literature.

The performance of the optimized ceramics prepared with ultra-low synthesis and sintering temperatures with two-step sintering (900 °C for 3 h and 4 h and 1280 °C for 6 h) and a second ball milling after synthesis is characterized by higher piezoelectric sensitivity (coupling and piezoelectric coefficients) and higher dielectric permittivity, together with and moderate dielectric losses after poling and lower mechanical quality factor than the ceramics here prepared at 1450 °C for 3 h. Table 5 also shows that the performance of these ceramics surpass the one of many other BCZT ceramics processed with higher temperatures. This takes place as a result of their chemical, crystallographic and microstructural homogeneity and absence of secondary phases.

## 4. Conclusions

A novel route of fabrication of BCZT piezoceramics, based on attrition milling in isopropanol, was developed. The efficiency of the attrition milling for 6 h in isopropanol is evidenced by the reduction of the temperature for the formation of the main perovskite structure, 854 °C in the unmilled powder to 582 °C for the 6 h milled powder.

The strong weight loss at the conventional synthesis conditions (1350 °C for 4 h) was drastically reduced by using an ultra-low thermal budget of 700 °C for 30 min. This leads to the formation of a majoritarian perovskite-type structure BCZT compound. However, in-depth analysis by XRD and SEM revealed that even for synthesis at 590 °C for 15 min, some secondary phases, mainly CaTiO_3_, are formed simultaneously to the BCZT compound.

The sintering temperature of 1280 °C was found optimum, well below those conventionally reported (>1400 °C), due to the high reactivity of the powder calcined at ultra-low temperature. A second attrition ball milling after synthesis improved the grain size of the sintered ceramic body, while having little effect on the content of secondary phases in the final ceramic. Sintering at 1280 °C for 4 h gives place to a better densification and final properties than sintering at 1300 °C for 4 h. However, for this ultra-low sintering conditions, the best properties obtained (d_33_ = 189 pC/N, k_p_ = 19%, Q_m_ = 197) are below the expected values, because of the persistence of secondary phases.

To enhance sinterability, two-step sintering was conducted from powder synthesized at 700 °C 2 h. By this method, single-phase perovskite BCZT ceramic with high piezoelectric sensitivity were obtained for sintering at 900 °C for 3 h and 900 °C 4 h, with final plateau of 1280 °C for 6 h. The best electromechanical properties achieved were d_33_ = 455 pC/N, k_p_ = 35%, Q_m_ = 155.

## Figures and Tables

**Table 1:** Rietveld analysis of the sintered ceramics after synthesis at 700 °C and 2 h under variable time and temperature using a two-step sintering.

Sintering Conditions	Cell Parameters (Å) c	Cell Parameters (Å) a	Tetragonal Distortion c/a	Main Perovskite Phase G.P.
900 °C/ 1 h + 1280 °C/2 h	4.0237	4.0030	1.005	P4mm BCZT
900 °C/ 1 h + 1280 °C/4 h	4.0306	4.0090	1.005	P4mm BCZT
900 °C/2 h + 1280 °C/4 h	4.0216	4.0011	1.005	P4mm BCZT
900 °C/2 h + 1280 °C/6 h	4.0143	3.9978	1.004	P4mm BCZT
900 °C/3 h + 1280 °C/6 h	4.0235	4.0099	1.003	Single Phase P4mm BCZT
900 °C/4 h + 1280 °C/6 h	4.0205	4.0030	1.004	Single Phase P4mm BCZT

**Table 2:** Some properties of the sintered ceramic disks, after synthesis at 700 °C for 30 min., under variable time and temperature using a single sintering plateau.

Properties\ Sintering Conditions	1150 °C 1 h (1 BM)	1260 °C 30 min (2 BM)	1280 °C 2 h (2 BM)	1280 °C 4 h (2 BM)	1300 °C 4 h (2 BM)
**Density (g/cc)**	4.34	4.60	5.02	4.78	4.41
**Resistance (MΩ)**	0.6	0.4	2	30	15
$\varepsilon \prime^{T}$**/tanδ** (at 1kHz) ^[1]^	1671 0.264	3396 0.543	2306 0.286	1903 0.159	2463 0.175
**d_33_ (pC/N)** ^[2]^	38	52	185	189	125

^[1]^ before poling; ^[2]^ from Berlincourt meter at 100 Hz.

**Table 3:** Some properties of the sintered ceramics after synthesis at 700 °C for 2 h under variable time and temperature using a two-step sintering method.

Properties/ Sintering	900 °C/1 h 1280 °C/2 h	900 °C/1 h 1280 °C/4 h	900 °C/2 h 1280 °C/6 h	900 °C/3 h 1280 °C/6 h	900 °C/4 h 1280 °C/6 h
**Density (g/cc)**	4.30	4.45	4.47	4.32	4.28
**Resistance (MΩ)**	5	8	9	1	2
$\varepsilon \prime^{T}$**/tanδ** (at 1 kHz) ^[1]^	2100 0.326	2014 0.282	2158 0.262	3052 0.511	2833 0.251
**d_33_ (pC/N) ^[2]^**	145	200	140	405	455

^[1]^ before poling; ^[2]^ from Berlincourt meter at 100 Hz.

**Table 4:** Some relevant material coefficients obtained from the Radial mode of resonance of the sintered ceramics after synthesis at 700 °C under variable sintering method. The complex material coefficients (P = P′ + iP′′) are given as real part (P′) and losses (piezoelectric and mechanical Q factor (P′/P′′) and dielectric tanδ (P′′/P′)). The data for the sample calcined at 1350 °C are also shown for comparison.

Properties\ Sintering Conditions	1150 °C 1 h (1 BM)	1280 °C 2 h	1280 °C 4 h	900 °C 1 h 1280 °C 4 h	900 °C 3 h 1280 °C 6 h	900 °C 4 h 1280 °C 6 h	1450 °C 3 h ^[2]^
** 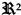 **	0.9996	0.9997	0.9995	0.9998	0.9999	0.9975	0.9964
**k_p_ (%)**	4.65	15.36	19.02	23.23	29.31	35.12	27.82
**N_p_(kHz.mm)**	2652	2740	2874	2897	2339	2559	2742
**d′_31_ (pC/N)**	−9.89	−36.3	−45.0	−55.7	−99.6	−108.8	−68.17
**Q_p_(d_31_)**	79	38	49	46	49	21	130
$\varepsilon \prime_{33}^{T}$ **/** **tanδ**	947 0.026	1458 0.029	1542 0.022	1498 0.021	1897 0.020	1797 0.078	1540 0.013
**g′_31_ (pC/N)**	−1.18	−2.81	−3.30	−4.20	−5.93	−6.81	−5.0
$c\prime_{11}^{p}$ **^[1]^** **(10^10^N m^−2^)**	7.36	8.71	8.93	8.41	5.40	6.57	8.40
**Q_m_**	208	162	197	188	120	155	157

^[1]^${c\prime }_{11}^{p}=$ s^E^_11_/s^E^_12_^[2]^ calcined at 1350 °C for 4 h.

**Table 5:** Comparison of the piezoelectric coefficient (d33) of different BCZT compositions prepared by different synthesis routes. SSR = solid-state route.

Synthesis Method	Synthesis T (°C)	Sintering T (°C)	d_33_ (pC/N)	Composition	Reference
**SSR**	1200	1450	365	BCZT0805	[32]
**SSR**	1100	1250	340	Li-modified BCZT0102	[40]
**SSR**	1350	1450 1500	620	BCZT1510	[8]
**SSR**	1250	1420	406	BCZT1510	[17]
**SSR**	1250	1400	410	BCZT1510	[21]
**SSR**	1250	1400	300	BCZT1010	[21]
**SSR**	1300	1500	330	BCZT1510	[10]
**SSR**	1200	1450	328	BCZT1610	[41]
**Mechano-activation**	900	1450	270	BCZT1510	[22]
**Sol-gel**	1000	1420	540	BCZT1510	[17]
**Pechini**	700	1275	390	BCZT1010	[16]
**Hydrothermal**	240	1300	164	BCZT1510	[18]

## Data Availability

The data presented in this study are available on request from the corresponding author.
